# Kritische Auseinandersetzung mit neuen Daten zur Prävalenz von Opioidgebrauchsstörungen bei Patienten mit chronischen Schmerzen in Deutschland

**DOI:** 10.1007/s00482-021-00582-1

**Published:** 2021-09-10

**Authors:** Johannes Just, F. Petzke, N. Scherbaum, L. Radbruch, K. Weckbecker, W. Häuser

**Affiliations:** 1 Department für Humanmedizin, Institut für Allgemeinmedizin und ambulante Gesundheitsversorgung (IAMAG), Universität Witten/Herdecke, Alfred-Herrhausen-Str. 50, 58448 Witten, Deutschland; 2 Schmerzmedizin, Klinik für Anästhesiologie, Universitätsmedizin Göttingen, Göttingen, Deutschland; 3 LVR-Klinikum Essen, Klinik für Psychiatrie und Psychotherapie, Kliniken der Universität Duisburg-Essen, Essen, Deutschland; 4 Helios Klinikum Bonn/Rhein-Sieg, Zentrum für Palliativmedizin, Akademisches Lehrkrankenhaus, Universität Bonn, Bonn, Deutschland; 5 Innere Medizin, Klinikum Saarbrücken, Saarbrücken, Deutschland; 6 Klinik für Psychosomatische Medizin und Psychotherapie der Technischen Universität München, München, Deutschland

**Keywords:** Chronische nichttumorbedingte Schmerzen, Opioidmissbrauch, Opioidabhängigkeit, Rezeptierte Opioide, Leitlinie, Chronic noncancer pain, Opioid misuse, Opioid dependence, Prescribed opioids, Guideline

## Abstract

**Hintergrund:**

Es gibt keine Opioidkrise in Deutschland. Neue Studien mit Nichttumorpatienten mit chronischen Schmerzen (CNTS) in Deutschland zeigen jedoch eine unerwartet hohe Prävalenz von Opioidgebrauchsstörungen nach Diagnostic and Statistical Manual for Psychiatric Diseases 5 (DSM-5).

**Ziel der Arbeit:**

Kritische Diskussion neuer Studienergebnisse zur Prävalenz von Opioidgebrauchsstörungen bei Schmerzpatienten in Deutschland.

**Material und Methoden:**

Selektive Literaturrecherche und multiprofessionelle Einordnung der Ergebnisse durch Expertenrunde (Schmerztherapie, Neurologie, Psychiatrie, Palliativmedizin, Allgemeinmedizin und Suchttherapie).

**Ergebnisse:**

Die Kriterien für die Diagnose „Opioidgebrauchsstörung“ des DSM‑5 sind auf Patienten mit CNTS nur eingeschränkt anwendbar, können aber für problematisches Verhalten sensibilisieren. Hierbei ist die Diagnose Opioidgebrauchsstörung nicht mit der Diagnose einer Substanzabhängigkeit nach ICD-10 gleichzusetzen, da die Diagnose nach DSM‑5 ein deutlich breiteres Spektrum abdeckt (mild, moderat, schwer). Risikofaktoren für eine Opioidgebrauchsstörung sind jüngeres Alter, depressive Störungen, somatoforme Störungen und hohe Opioidtagesdosen. Die interdisziplinäre Leitlinie zur Langzeitanwendung von Opioiden bei chronischen nichttumorbedingten Schmerzen (LONTS) enthält Empfehlungen, welche das Risiko für eine Opioidgebrauchsstörung reduzieren sollen.

**Diskussion:**

Eine Anpassung der DSM-5-Diagnosekriterien der Opioidgebrauchsstörung an die besondere Situation von Patienten mit CNTS und eine Validierung dieser Kriterien könnte helfen, in der Zukunft genauere Daten zu Opioidgebrauchsstörungen von Patienten mit chronischen Schmerzen in Deutschland zu erheben. Verordner sollten für diese Problematik sensibilisiert werden, ohne die Patienten zu pathologisieren oder gar zu stigmatisieren. Weitere Forschung zur Einordnung dieses bisher unterschätzten Phänomens ist notwendig.

## Abstract (en)

**Background:**

There is no opioid crisis in Germany. However, new studies involving patients with chronic noncancer pain (CNCP) in Germany show an unexpectedly high prevalence of opioid use disorder according to DSM‑5 (Diagnostic and Statistical Manual for Psychiatric Diseases).

**Objectives:**

Critical discussion of new study results on the prevalence of opioid use disorder in CNCP patients in Germany.

**Materials and methods:**

Selective literature search and multiprofessional classification of results by an expert panel (pain therapy, neurology, psychiatry, palliative medicine, general medicine and addiction therapy).

**Results:**

The DSM‑5 criteria for the diagnosis of “opioid use disorder” have limited applicability to patients with CNCP, but may raise awareness of problematic behavior. The diagnosis of opioid use disorder is not the same as the diagnosis of substance dependence according to ICD-10, as the DSM‑5 diagnosis covers a much broader spectrum (mild, moderate, severe). Risk factors for opioid use disorder include younger age, depressive disorders, somatoform disorders, and high daily opioid doses. The interdisciplinary guideline on long-term opioid use for CNCP (LONTS) includes recommendations intended to reduce the risk for opioid use disorder.

**Conclusion:**

An adaptation of the DSM‑5 diagnostic criteria of opioid use disorder to the specific situation of CNCP patients and a validation of these criteria could help to collect more accurate data on opioid use disorders of patients with chronic pain in Germany in the future. Prescribers should be sensitized to this problem without pathologizing or even stigmatizing patients. Further research is needed to classify this previously underestimated phenomenon.

## Der Stellenwert von Opioiden bei chronischen Schmerzen

Opioidhaltige Analgetika gehören zur WHO-Liste der „essenziellen Medikamente“. Opioidhaltige Schmerzmittel haben einen hohen Stellenwert in der Tumorschmerztherapie und Palliativmedizin [26]. Der Stellenwert von Opioiden bei chronischen Nichttumorschmerzen (CNTS) ist jedoch umstritten [2]. Opioidhaltige Schmerzmittel sind bezüglich der Schmerzreduktion nichtopioidhaltigen Schmerzmitteln und nichtmedikamentösen Therapieoptionen nicht überlegen [3]. Dennoch entfallen circa drei Viertel aller Opioidverschreibungen in Deutschland auf Patienten mit CNTS [19, 22].

## Gibt es eine Opioidepidemie in Deutschland?

In der aktualisierten Leitlinie zur Langzeitanwendung von Opioiden bei chronischen nichttumorbedingten Schmerzen (LONTS) besteht Konsens, dass es in Deutschland keinen opioidbezogenen Gesundheitsnotstand („Opioidkrise“ oder „Opioidepidemie“) wie in den USA gibt [11]. In den letzten Jahren sind jedoch mehrere Studien einer deutschen Arbeitsgruppe erschienen, welche die Häufigkeit einer Opioidgebrauchsstörung sowie das Risiko von Opioidmissbrauch bei Patienten mit chronischen Schmerzen untersuchten, jedoch in der aktuellen LONTS-Leitlinie nicht berücksichtigt wurden [13, 15, 16]. Die Ergebnisse dieser Studien wurden aktuell im Rahmen eines Leserbriefs in *Der Schmerz* zum Anlass genommen, diese Aussage von LONTS infrage zu stellen und als „Verharmlosung“ zu bezeichnen [25].

Im Folgenden sollen die Kriterien einer Opioidgebrauchsstörung und die Ergebnisse der erwähnten Studien dargestellt und aus multiprofessioneller Perspektive diskutiert werden.

## Aktuelle Studien zur Prävalenz von Gebrauchsstörungen von rezeptierten Opioiden durch Patienten mit chronischen Schmerzen in Deutschland

Im Juli 2020 erschien eine Analyse zu Gebrauchsstörungen von rezeptierten Opioiden durch Patienten mit chronischen Schmerzen in Deutschland, die auf den Daten des Suchtsurvey 2015 basiert [15]. Unter dem Titel „Rate of opioid use disorder in adults who received prescription opioid pain therapy—A secondary data analysis“ beschreiben Just und Kollegen die Prävalenz von Opioidgebrauchsstörungen nach den Kriterien des DSM‑5 in einer repräsentativen deutschen Kohorte (Kriterien für eine Opioidgebrauchsstörung siehe Tab. 1). Von 9204 Studienteilnehmern hatten *n* = 275 (3,5 %) im Jahr 2015 Opioide verschrieben bekommen. Davon erfüllten *n* = 54 (21,2 %) die im Survey erfragten Diagnosekriterien für eine Opioidgebrauchsstörung nach DSM‑5. Im Detail erfüllten 14,7 % die Kriterien für eine leichte Opioidgebrauchsstörung, weitere 3,5 % für eine mittelgradige und 2,9 % für eine schwere Opioidgebrauchsstörung. Das Vorliegen einer Opioidgebrauchsstörung bei Opioidverschreibung war häufiger assoziiert mit psychiatrischen Erkrankungen, depressiven Störungen und dem Vorliegen nicht erklärbarer körperlicher Symptome und weniger häufig assoziiert mit dem Gebrauch nichtopioidhaltiger Analgetika. In einer kleineren, nicht repräsentativen Erhebung der DSM-5-Kriterien bei Schmerzpatienten, die sich in schmerztherapeutischer Betreuung befanden (vier Praxen) und verschreibungspflichtige Opioide bereits länger als 6 Monate einnahmen, fiel die Rate an Opioidgebrauchsstörung mit 26,5 % (leichtgradig: 17,2 % | mittelgradig: 4,4 % | hochgradig: 4,9 %) sogar noch etwas höher aus [16].

**Table 1:** 

Kriterien der Opioidgebrauchsstörung: (Definition: problematisches Opioidkonsummuster, dass erhebliche Beeinträchtigungen oder Stress verursacht)	Rate positiver Antworten (%)	
	Studie 1 [16]	Studie 2 [15]
1. Die Substanz wird häufig in größeren Mengen oder über einen längeren Zeitraum eingenommen als ursprünglich beabsichtigt	13,5	9,4
2. Es besteht ein anhaltendes Verlangen oder ein nicht erfolgreicher Versuch, den Substanzkonsum zu kontrollieren oder zu reduzieren	19,8	15,0
3. Ein großer Teil der Zeitspanne wird damit verbracht, die Substanz zu besorgen, zu konsumieren oder sich von den Auswirkungen des Konsums zu erholen	7,6	5,2
4. Es besteht ein Craving (starkes Verlangen) oder ein Druck, die Substanz zu konsumieren	14,8	5,7
5. Wiederholter Substanzkonsum führt zum Versagen bei der Erfüllung wichtiger Aufgaben (Rollenverpflichtungen im Beruf, in der Schule oder zu Hause)	11,0	20,8
6. Es besteht ein anhaltender Substanzkonsum ungeachtet der bestehenden oder wiederholt auftretenden sozialen oder interpersonellen Probleme, die durch die Effekte des Substanzkonsums verursacht oder verstärkt werden	4,2	6,3
7. Relevante soziale, berufliche oder freizeitbezogene Aktivitäten werden wegen des Substanzkonsums aufgegeben oder reduziert	14,4	11,5
8. Die Substanz wird wiederholt in Situationen konsumiert, in denen dies körperlich gefährlich ist	5,9	14,9
9. Der Substanzkonsum wird ungeachtet des Wissens um bereits bestehende oder wiederauftretende physische oder psychische Probleme fortgesetzt, die sehr wahrscheinlich durch den Substanzkonsum verursacht oder verstärkt werden	8,9	3,6

2–3 Kriterien: mild, 4–5 Kriterien: moderat, ≥ 6 Kriterien: schwere Störung

Kriterien 10 (Toleranzentwicklung) und 11 (Entzugssymptome) wurden gemäß Diagnosekriterien nicht gezählt, da die untersuchten Patienten unter ärztlicher Therapiekontrolle waren

In einer weiteren Studie in Hausarztpraxen konnte bei 31,5 % der untersuchten Patienten, die verschreibungspflichtige Opioide bereits länger als 6 Monate einnahmen, ein „stark erhöhtes Risiko für Opioidmissbrauch“ festgestellt werden [13]. Das hier verwendete Screeninginstrument, der COMM Score® (Inflexxion Inc., Waltham, MA, USA), ist für die Nutzung in einem primärärztlichen Setting validiert und ist geeignet, Patienten mit einem „stark erhöhten Risiko für Opioidmissbrauch nach DSM-IV“ zu identifizieren. Die Testgüte in Bezug auf die DSM-IV-Diagnose „Opioidmissbrauch“ liegt dabei bei einer Sensitivität von 77 % sowie einer Spezifität von 77 % [20].

Eine Schwäche aller drei zitierten Studien ist die fehlende Erhebung der morphinäquivalenten Tagesdosis der genutzten Opioide sowie für die Studien [15] und [16] das Fehlen einer ärztlichen Kontrolle der erhobenen Diagnosekriterien.

## Widersprüche zwischen Prävalenz und Mortalität

### Widersprüche zwischen Prävalenz und Mortalität

Die hohe Rate von Opioidgebrauchsstörungen bei Patienten mit CNTS in Deutschland in beiden Studien steht scheinbar im Widerspruch zu der niedrigen Drogensterblichkeit und der niedrigen Rate von opioidabhängigen Personen in Deutschland. Während die jährliche Drogensterblichkeit in den USA 20/100.000 Einwohner beträgt, liegt sie in Deutschland lediglich bei 1,6/100.000 Einwohner. Auch die Prävalenz der Opiatabhängigkeit ist in Deutschland mit 0,2 % deutlich niedriger als in den USA mit 4 % [1, 18]. Ebenso gibt es in Deutschland keine Berichte über Personen des öffentlichen Lebens, die von medikamentenassoziierten Todesfällen betroffen sind, ganz anders als in den USA, wo seit Jahren vermehrt berühmte Schauspieler, Musiker und Sportler an Überdosen verschreibungspflichtiger Opioide versterben [14]. Aber sowohl solche Fallbeschreibungen als auch die Todesursachenstatistik sind wegen Unterschieden in der Erhebungsmethodik nur begrenzt aussagekräftig und vergleichbar.

### Gründe für die hohe Prävalenz von Opioidgebrauchsstörungen in den vorgestellten Studien

Aus unserer Sicht lassen sich die Differenzen zwischen Prävalenz und Mortalität aus unterschiedlichen Perspektiven erklären:

1. *Definition und diagnostischer Rahmen im DSM-5:* „Opioidgebrauchsstörung“ ist definiert als „ein problematisches Opioidkonsummuster, dass erhebliche Beeinträchtigungen oder Stress verursacht“. Das Ziel dieser neuen Definition war es, stigmatisierte Begriffe wie Sucht und Missbrauch zu vermeiden, aber auch das Spektrum opioidbezogener Störungen differenzierter abzubilden. Daher korrespondieren nur die Diagnosekategorien „mittelgradige“ und „schwere“ Opioidgebrauchsstörung mit den Missbrauchs- und Suchtkriterien in DSM-IV und ICD-10 [6]. Somit umfasst die „Opioidgebrauchsstörung“ eine größere Patientengruppe, von der 6,4 % Patienten mit mittel- bis schwergradiger Opioidgebrauchsstörung dem bisherigen Verständnis von Missbrauch/schädlichem Gebrauch und Abhängigkeit, wie es z. B. in der ICD-10 formuliert ist, entsprechen, während die 14,7 % mit einer leichten Opioidgebrauchsstörung Patienten sind, die aufgrund eines erhöhten Risikos ggf. lediglich eines intensivierteren Follow-ups bedürfen.
2. *Eine möglicherweise unzureichende Trennschärfe zwischen einer Opioidgebrauchsstörung und unzureichend behandelten Schmerzen im DSM-5:* Es ist denkbar, dass bei Schmerzpatienten einzelne Diagnosekriterien im Rahmen des DSM‑5 falsch interpretiert werden. So könnten zum Beispiel Kriterien wie „Opioide werden oft in größerer Menge oder über einen längeren Zeitraum als vorgesehen eingenommen“ oder „Verlangen oder ein starker Wunsch, Opioide zu nehmen“ auch Ausdruck von persistierenden oder eskalierenden Schmerzen sein und nicht Ausdruck einer Opioidgebrauchsstörung [10]. Eine Übersicht qualitativer Studien legte zudem nahe, dass Patienten auch unter ausreichender Opioidtherapie eine intensive Ambivalenz zwischen erlebter Wirkung, Nebenwirkungen und dem Gefühl der Notwendigkeit der Einnahme, als einer Form von Abhängigkeit, erleben [21]. Die Diagnose Opioidgebrauchsstörung ist definiert als ein problematisches Muster des Opioidkonsums, das erhebliche Beeinträchtigungen oder Stress verursacht – ein solches problematisches Konsummuster kann u. U. auch bei schwer zu beeinflussenden Schmerzen bestehen. Elander und Kollegen zeigten bei Patienten mit chronischen Schmerzen bei Sichelzellanämie eine deutliche Reduktion der Häufigkeit der Diagnose Opioidmissbrauch und -abhängigkeit nach DSM-IV, wenn für den Umstand einer schmerzbedingt positiven Antwort korrigiert wurde [8]. Daher ist weitere Forschung nötig, um zu prüfen, ob einzelne Diagnosekriterien des DSM‑5 bei chronischen Schmerzpatienten anders oder sogar überhaupt nicht genutzt werden sollten.
3. *Die besondere wirtschaftliche, gesellschaftliche und soziale Situation in den USA:* Wahrscheinlich spielen bei der Entwicklung des nationalen Gesundheitsnotstands in den USA („Opioidkrise“ oder „Opioidepidemie“) gesellschaftliche Faktoren eine große Rolle, die nicht auf andere Staaten generalisiert werden können. Daher ist es möglich, dass es auch in Deutschland bei Patienten mit Verschreibung von Opioidanalgetika Opioidgebrauchsstörungen gibt, ohne dass sich daraus ein nationaler Gesundheitsnotstand entwickeln müsste. Die Ökonomen Anne Case und Angus Deaton beschreiben in ihrem 2020 erschienenen Buch *Deaths of Despair* die Krise der amerikanischen Mittelschicht und deren verheerenden Einfluss auf die Lebenserwartung in den USA. Die USA sind das einzige industrialisierte Land der Welt, in dem die Lebenserwartung im 21. Jahrhundert gesunken ist. Hauptsächlich lässt sich dieser Effekt auf eine Gruppe zurückführen, die Case und Deaton als „Verzweiflungstote“ bezeichnen, die Opfer von Selbstmord, Alkoholabhängigkeit und Opioidüberdosierungen. Dieser Effekt findet sich in den USA allerdings nur in einer Bevölkerungsschicht in dieser Deutlichkeit: der weißen, nichtakademischen Mittelschicht. Case und Deaton erklären diese „Verzweiflungstoten“ durch Arbeitslosigkeit und Kaufkraftverlust in dieser Schicht, die besonders stark durch die Abwanderung und den Abbau von Arbeitsstellen in der produzierenden Industrie betroffen ist. Da solidarische Sicherungssysteme in den USA nur minimal ausgeprägt sind und von Betroffenen teilweise sogar ideologisch abgelehnt werden, konnte dieser soziale und ökonomische Abstieg nicht abgefedert werden [4]. Als weiterer USA-spezifischer Faktor ist das in der Vergangenheit sehr aggressive Marketingverhalten einzelner Opioidhersteller zu nennen. Neben dem direkten Konsumentenmarketing (Werbung am Patienten, in Deutschland nicht erlaubt) trugen auch gezielte Fehlbehauptungen zur Entwicklung der Opioidkrise in den USA bei [9]. Zu den Fehlbehauptungen über ihr Produkt „Oxycontin“, zu denen sich die Firma Purdue Pharma bekannt hat, gehört unter anderem, dass es nicht euphorisierend wirke und keine Gewöhnungs- oder Entzugseffekte auftreten. Im Zusammenhang von hohen Vergleichszahlungen meldete die Firma 2019 Konkurs an [5, 27].
4. *Die besondere wirtschaftliche, gesellschaftliche und soziale Situation in Deutschland: *Das Vorhandensein und die hohe Qualität sozialer und solidarischer Sicherungssysteme in Deutschland ist wahrscheinlich ein wichtiger Einflussfaktor, der die Entwicklung von der Opioidgebrauchsstörung zur Opioidabhängigkeit und zum Tod durch Überdosis hemmt. Qualitativ hochwertige ambulante und stationäre Versorgungsangebote sind für Menschen in Deutschland meist niederschwellig erreichbar. Hierzu zählt die primärärztliche Grundversorgung, aber auch spezialisierte Angebote wie Schmerztherapie, suchtmedizinische Grundversorgung, Psychotherapie und spezialisierte psychiatrische Angebote [7].

## Prävention

Es bleibt die kommunikative Herausforderung, wie eine angemessene Prävention und Erkennung von Opioidgebrauchsstörungen in Deutschland erfolgen kann, ohne dadurch unnötige Sorgen vor Opioidnebenwirkungen bei Ärzten und Patienten zu schüren.

Der Schlüssel zur erfolgreichen Prävention liegt zum einen in der richtigen Indikation, Dosierung und Therapiedauer und zum anderen im frühzeitigen Erkennen potenziell gefährdeter Patienten. Dies beinhaltet laut LONTS-Leitlinie konkret: a) das Screening auf psychische und substanzbezogene Störungen und gegebenenfalls eine fachpsychotherapeutische/psychiatrische Vorstellung vor Beginn einer Therapie mit Opioiden, b) das Beachten von Kontraindikationen (z. B. psychische Störungen mit Leitsymptom Schmerz), c) das Festlegen von Therapiezielen, d) die Beendigung der Therapie, wenn die Therapieziele nicht erreicht sind, und e) regelmäßige Evaluation der Wirksamkeit und Nebenwirkungen inklusive Hinweise missbräuchlicher Verwendung [11].

## Erkennen einer Opioidgebrauchsstörung

Die bisher für die deutsche Bevölkerung beschriebenen Risikoindikatoren für eine Opioidgebrauchsstörung bzw. Missbrauch/Abhängigkeit von rezeptierten Opioiden sind:

- Jüngeres Lebensalter: OR 0,96 (95 % CI 0,95; 0,97; [19]) sowie OR 0,96 (95 % CI 0,94; 0,99; [16])
- Somatoforme Schmerzstörung: OR 1,92 (95 % CI 1,18; 23,16) und OR 1,89 (95% CI 1,56; 2,28; [12, 19]) bzw. unklare körperliche Beschwerden OR 2,68 (95 % CI 1,14; 6,31; [15])
- Depressive Störungen: OR 2,26 (95 % CI 1,37; 6,58), OR 2,52 (95 % CI 2,12; 3,00) und OR 2,69 (95 % CI 1,13; 6,38; [12, 15, 19])
- Hochdosis-LTOT (> 120 mg morphinäquivalente Tagesdosis) 1,81 (95 % CI 1,44; 2,27; [12])
- Psychiatrische Diagnose OR 4,12 (95 % CI 1,36; 12,43; [15])

Eine Vorgeschichte von einer substanzbezogenen Störung, z. B. Alkoholabhängigkeit, ist ein, im internationalen Setting, häufig nachgewiesener und als sehr relevant eingestufter Risikofaktor, der in Deutschland bisher nicht nachgewiesen wurde, aber mutmaßlich auch hier eine große Rolle spielt [23, 24]. Die LONTS-Leitlinie stellt zudem eine Aufzählung auffälliger Verhaltensmuster einer missbräuchlichen/abhängigen Verwendung zur Verfügung, welche in den diagnostischen Kriterien von DSM-IV, DSM‑5, ICD-10 und ICD-11 nicht enthalten sind, deren Anwendung klinisch aber sehr hilfreich sein kann (Tab. 2, [11]).

**Table 2:** 

Nichtspezifische Hinweise für Fehlgebrauch	Nichtspezifische Hinweise für Missbrauch/Abhängigkeit	Spezifischere Hinweise auf Abhängigkeit
▪Einnahme trotz geringer bis fehlender Wirksamkeit ▪Ein Fehlgebrauch, der trotz Aufklärung, Absprache und engerer ärztlicher Anbindung anhält, kann als Hinweis auf einen schädlichen Gebrauch/Abhängigkeit gewertet werden ▪Wechselnde Schmerzlokalisationen, multilokuläre Ausbreitung (Generalisierung) der Schmerzen, Transformation des Primärschmerzes unter laufender Therapie ▪Opioidinduzierte Hyperalgesie (Tendenz zur Schmerzausbreitung, Erhöhung der Schmerzempfindlichkeit und Opioidresistenz)	▪Hoher Ruheschmerz sowie Diskrepanz zwischen Schmerzangabe und Verhalten ▪Fordern eines bestimmten Opioids, speziell von kurz wirksamen und/oder schnell anflutenden Opioiden ▪Opioideinnahme überwiegend zur Symptomlinderung (Disstress, Unruhe, Angst, Depression, Schlafstörung) ▪Nicht abgesprochene Dosiserhöhungen ▪Drängen auf Dosiserhöhung ohne Verbesserung der Symptome/Funktion oder trotz Zunahme der Nebenwirkungen ▪Wiederholte Unzuverlässigkeiten (Unpünktlichkeit, Nichterscheinen) oder mangelnde Compliance ▪Verschwiegene Einnahme von Substanzen mit Suchtpotenzial (Diskrepanzen beim Drug-Monitoring) ▪Drängen auf Verschreibung weiterer psychotroper Substanzen ▪Veränderung der verabredeten Einnahmeintervalle, eigenständige Anpassung nach Bedarf ▪Abwehr von Therapieänderungen ▪Wesensveränderungen unter der Therapie (z. B. Impulskontrollstörungen) und andere neue psychiatrische Symptome ▪Missbrauch anderer Substanzen zu psychotropen Zwecken ▪Zunahme von Depressivität, Angststörung, Albträumen unter der Therapie	▪Anhaltender Widerstand gegen Änderungen der Medikation trotz Wirkungslosigkeit und/oder Symptomen einer ärztlich unerwünschten psychotropen Wirkung (Euphorie, Sedierung, Angstlinderung) ▪Anhaltender Widerstand gegen Änderungen der Medikation trotz psychotroper (zumeist dosisabhängiger) Nebenwirkungen (Müdigkeit, Antriebslosigkeit, Konzentrationsstörungen) ▪Injektion oraler/transdermaler Verabreichungsformen ▪Intravenöse und orale Anwendung transdermaler Systeme ▪Rezeptfälschungen ▪Stehlen/Borgen von Opioiden ▪Nichtplausibles Horten der Medikamente ▪Verheimlichter/abgestrittener Bezug durch andere Ärzte ▪Verschwiegener Konsum von weiteren psychotropen Substanzen einschließlich anderer Opioide ▪Häufiger Verlust von Rezepten ▪Fordern eines parenteralen Verabreichungswegs ▪Handel von Opioiden mit Dritten ▪Kontrollverlust (z. B. wiederholte Episoden von Dosiserhöhungen oder zunehmend bedarfsgesteuerte Einnahme trotz klarer Absprache/Warnung, deutliche unmittelbare negative Folgen der Medikamenteneinnahme im privaten und sozialen Umfeld) ▪Zwanghafter Gebrauch

## Klinische Evaluation potenziell betroffener Patienten

Bisher gibt es kein ausreichend validiertes und geeignetes Screeninginstrument für das Vorliegen opioidassoziierter Probleme, das in der Breite der Patientenversorgung in Deutschland uneingeschränkt empfohlen werden könnte. Die besondere Stärke der DSM-5-Diagnosekriterien für eine Opioidgebrauchsstörung liegt aus unserer Sicht darin, dass zum einen ein dynamisches Spektrum abgebildet wird (leicht, mittel, schwer), und zum anderen stigmatisierende Begriffe wie Abhängigkeit und Sucht vermieden werden. Andererseits gibt es berechtigte Zweifel bezüglich der Anwendbarkeit einzelner Kriterien bei Patienten mit chronischen Schmerzen. Daher kann die klinische Nutzung aktuell nicht uneingeschränkt empfohlen werden, zumal in Deutschland die ICD-10/11 und nicht der DSM‑5 den diagnostischen Codierungsstandard darstellt. Somit stellt die aufmerksame klinische Beobachtung des Patienten unter anderem unter Berücksichtigung der genannten Risikoindikatoren und auffälliger Verhaltensmuster den Goldstandard der Erkennung betroffener Patienten dar. Ein weiterer zentraler Teil der Evaluation sollte immer die Frage sein, ob die betroffenen Schmerzpatienten aktuell mehr unter der Wirkung und Nebenwirkung der eingenommenen Opioide leiden, als sie durch eine Schmerzreduktion profitieren. Dabei gilt es zu prüfen, welche weiteren Maßnahmen der multimodalen Schmerztherapie noch ausgeschöpft werden können. Bei gegebener Indikation sind auch Maßnahmen zur Verbesserung der problematischen Anwendung (Edukation, engmaschigere Betreuung, Vereinbarung gemeinsamer Regeln zur Verordnung) sinnvoll. Erst wenn wiederholt Probleme mit Entzugssymptomen oder problematischem Verhalten des Patienten auftreten, ist eine suchtmedizinische/suchtpsychiatrische Vorstellung/Mitbehandlung dringend zu erwägen [11, 17].

## Fazit für die Praxis

- Mittel- bis schwergradige Opioidgebrauchsstörungen nach DSM‑5 zeigen eine gute Korrelation mit den bekannten Diagnosen „Abhängigkeit“ sowie „Missbrauch“ bzw. „schädlicher Gebrauch“ in DSM-IV und ICD-10. Die Prävalenz in Deutschland liegt wahrscheinlich im höheren einstelligen Bereich.
- Patienten jüngeren Alters mit depressiven Störungen, mit somatoformen Störungen und hohen Opioidtagesdosen sind besonders gefährdet, Opioidgebrauchsstörungen bzw. Missbrauch/Abhängigkeit zu entwickeln.
- Da Zweifel daran bestehen, ob alle Diagnosekriterien von DSM-IV, DSM‑5 und ICD-10/11 sinnvoll bei Patienten mit CNTS angewendet werden können, brauchen wir weitere Forschung zur Anwendbarkeit der genannten Diagnosekriterien.
- Das aufmerksame ärztliche Gespräch unter Beachtung bekannter Risikofaktoren und auffälligen Verhaltens ist aktuell der diagnostische Goldstandard zur Erkennung von Patienten mit opioidassoziierten Problemen.
- Die Leitlinien „LONTS Update“ und „Medikamentenbezogene Störung“ bieten Hinweise zum weiteren Umgang mit betroffenen Patienten.
